# Tough, Instant, and Repeatable Adhesion of Self‐Healable Elastomers to Diverse Soft and Hard Surfaces

**DOI:** 10.1002/advs.202105742

**Published:** 2022-02-20

**Authors:** Ke Li, Xingjie Zan, Chen Tang, Zhuangzhuang Liu, Jianghuan Fan, Gang Qin, Jia Yang, Wei Cui, Lin Zhu, Qiang Chen

**Affiliations:** ^1^ Wenzhou Institute University of Chinese Academy of Sciences Wenzhou 352001 China; ^2^ School of Materials Science and Engineering Henan Polytechnic University Jiaozuo 454000 China; ^3^ Oujiang Laboratory (Zhejiang Lab for Regenerative Medicine, Vision and Brain Health) Wenzhou 352001 China; ^4^ College of Polymer Science and Engineering State Key Laboratory of Polymer Materials Engineering Sichuan University Chengdu 610065 China

**Keywords:** adhesion, chain mobility, elastomer, energy dissipation, soft electronics

## Abstract

Repeatability and high adhesion toughness are usually contradictory for common polymer adhesives. Repeatability requires temporary interactions between the adhesive and the substrate, while high adhesion toughness is usually achieved by permanent bonding. Integrating these two features into one adhesive system is still a daunting challenge. Here, the development of a series of viscoelastic elastomers composed of a soft and hard segment is reported, which exhibit tough, instant, yet repeatable adhesion to a variety of soft and hard surfaces. Such a combination of mutually exclusive properties is attributed to the synergy of high mobility of polymer chains and massive viscoelastic dissipation of the elastomers around the interface. By optimizing the relaxation time and mechanical dissipation, the resulting adhesives can achieve a tough yet repeatable adhesion toughness above 2000 J m^−2^, superior to the best‐in‐class commercial adhesives. Numerous acrylate monomers are proven applicable to the preparation of such adhesives, validating the universality of the fabrication method. The application of these elastomers as adhesive and protective layers in soft electronics by virtue of their universal and tough adhesion to various soft and hard substrates is also demonstrated.

## Graphical abstract

Copolymerizing acrylate monomers with contrasting glass transition temperatures of their homopolymers results in an elastomer with a glass transition temperature that is slightly below room temperature. Due to high polymer chain mobility and energy dissipation, the viscoelastic elastomer shows tough, instant, and repeatable adhesion to diverse soft and hard surfaces.

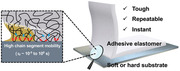

## 1 Introduction

Elastomer adhesives are indispensable in a large number of applications, including, but not limited to, artificial skins,^[^ ^1^, ^2^, ^3^, ^4^ ^]^ sealants,^[^ ^5^, ^6^, ^7^, ^8^ ^]^ and wearable electronics.^[^ ^9^, ^10^, ^11^, ^12^, ^13^, ^14^ ^]^ Unlike hard adhesives based on plastics or resins, elastomer adhesives are capable of conforming with the deformation of adherends, for instance, body parts, enabling them to be utilized in numerous emerging fields such as bio‐interfacial electrodes.^[^ ^15^, ^16^, ^17^, ^18^ ^]^ Realizing tough adhesion of the elastomer adhesives to diverse surfaces, either hard or soft, is crucial for developing soft devices with a high level of complexity.^[^ ^19^, ^20^ ^]^ However, achieving tough adhesion often comes at the expense of recyclability.^[^ ^21^ ^]^ That is, tough adhesion is usually permanent,^[^ ^22^, ^23^, ^24^ ^]^ and the elastomers can never be removed even when the devices reach their lifespan. Currently, a central challenge for elastomer adhesives is to combine reusability and tough adhesion, which allows environmental‐friendly recycling of devices without hampering their proper functioning during use.

Existing elastomer adhesives can be classified into two main types: temporary and permanent. Neither of these two types can perfectly integrate tough adhesion and reusability due to their intrinsic drawbacks. Temporary elastomer adhesives can directly adhere to the solid surfaces through reversible interactions, as exemplified by Van der Waals interaction,^[^ ^25^ ^]^ hydrogen bonding,^[^ ^26^ ^]^ hydrophobic interaction,^[^ ^27^ ^]^ reversible covalent bonds,^[^ ^28^, ^29^ ^]^ and polymer entanglements,^[^ ^30^ ^]^ etc. Such adhesives usually show rapid, immediate, and repeatable adhesion, whereas the adhesion toughness is low because of limited hysteresis of the bulk during interfacial separation. On the other hand, extremely strong bonding can be achieved by exploiting permanent links to connect substrates and elastomer adhesives.^[^ ^22^ ^]^ For example, surface modification is often utilized to form irreversible covalent bonds between the elastomers and adherends, enabling the adhesives to sufficiently dissipate energy during interfacial separation.^[^ ^31^ ^]^ Physical ways such as topological adhesion (a sort of mechanical interlocking) are also proven valid to form a permanent interface between the elastomer adhesive and substrates.^[^ ^32^ ^]^ However, permanent adhesion means that a portion of the adhesive inevitably remains on the surface of substrates during removal, which makes it impossible to recycle the products. All in all, combining reusability and tough adhesion has always been a daunting challenge for elastomer adhesives.

Theoretically, the adhesion toughness during peeling (*Γ*) between an adhesive and a substrate can be expressed as *Γ* = * Γ*_i_+*Γ*_d_,^[^ ^21^, ^33^ ^]^ where *Γ*_i_ is the intrinsic work of adhesion that relates to the interfacial interaction and *Γ*_d_ is the mechanical dissipation contributed by the adhesive that is highly deformed around the interface.^[^ ^34^, ^35^ ^]^ Therefore, simultaneously achieving repeatable and tough adhesion requires a synergy of reversible interfacial interaction and high energy dissipation of the material near the interface upon detachment. An ideal candidate is a viscoelastic material, which on one hand, possesses a short relaxation time, and on the other hand, exhibits massive hysteresis upon loading due to viscous dissipation.^[^ ^36^ ^]^ The short relaxation time results in high mobility of polymer chains, enabling repeatable adhesion via Van der Waals interactions. Meanwhile, the viscoelastic material can dissipate a significant amount of energy during interfacial separation, leading to a high *Γ*_d_ for achieving tough adhesion.

Based on the above considerations, here, we report a simple elastomer system that can simultaneously show instant, tough, yet repeatable adhesion to a variety of soft and hard surfaces. The key to combining these contradictory properties is to copolymerize acrylate monomers with contrasting glass transition temperatures (*T*_g_) of their homopolymers, resulting in a copolymer with a *T*_g_ slightly below room temperature. The resultant viscoelastic elastomer, on the one side, has a short relaxation time, and on the other side, is highly energy dissipative. Such a synergy enables the elastomer to show repeatable yet tough adhesion by virtue of its high mobility of polymer chains and massive hysteresis during interfacial separation. The tough adhesion, despite above 2000 J m^−2^ (90° peeling test), can be facilely repeated over 100 attach/detach cycles, with inappreciable loss in the adhesion toughness. Meanwhile, the elastomer is self‐recoverable and healable, demonstrating excellent durability. Creating viscoelastic elastomers for tough yet repeatable adhesion to various soft and hard surfaces is proven valid for a series of acrylate monomers, indicating the universality of the strategy. Finally, we show the facile assembly of a multilayer resistive sensor due to the tough adhesion between the elastomer and soft substrates, the components of which can also be on‐demand detached and recycled by virtue of the repeatable adhesion. This work provides a simple way to develop strong adhesives that realize tough and repeatable adhesion to diverse surfaces, which holds great potential for cost reduction of recycling hybrid devices in industrial fields.

## 2 Results and Discussion

### 2.1 Formation and Mechanical Properties

**Figure 1:**
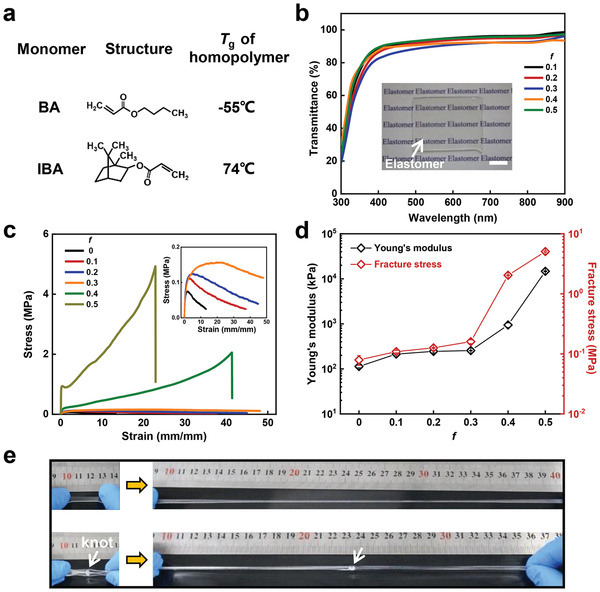
a) The abbreviation and chemical structures of typical monomers for the preparation of viscoelastic elastomers. Butyl acrylate is designated as BA, and isobornyl acrylate is designated as IBA. The *T*_g_ of corresponding homopolymers of the monomers are listed. b) Transmittance of P(BA*‐co‐*IBA) elastomers with varied molar ratios of IBA, *f*, which is defined as *f* = *M*_IBA_/(*M*_IBA_+*M*_BA_). The inset shows the high transparency of a piece of P(BA‐*co*‐IBA)_0.4_ elastomer (thickness of 1 mm), which is put on a piece of background paper. The scale bar represents 10 mm. c) Tensile stress–strain curves of P(BA*‐co‐*IBA) elastomers with varied *f*. The testing velocity is 100 mm min^−1^, corresponding to a strain rate of 0.14 s^−1^. d) Young's modulus (black points) and fracture stress (red points) of P(BA‐*co*‐IBA) elastomers with varied *f*. e) Demonstration of the extraordinary mechanical properties of the *f* = 0.4 elastomer by stretching and knotted stretching.

As a typical example, we first show a combination of two monomers with contrasting *T*_g_ of corresponding homopolymers to fabricate viscoelastic elastomer adhesives (**Figure** **1**a). The homopolymer of the monomer butyl acrylate (BA) possesses a *T*_g_ that is far below room temperature, which acts as a soft segment when forming copolymers with other monomers. In contrast, the homopolymer of isobornyl acrylate (IBA) is remarkably stiff due to its considerably high *T*_g_, playing the role of the hard segment. Since the two monomers are both low‐viscosity liquid, a series of P(BA*‐co‐*IBA) elastomers can be facilely prepared by a one‐step free‐radical copolymerization. The resulting samples are distinguished by *f*, which is defined as the molar ratio of IBA by *f* = *M*_IBA_/(*M*_IBA_+*M*_BA_). Wide angle X‐ray scattering (WAXS) and small angle X‐ray scattering (SAXS) measurements indicate that the elastomers have an amorphous structure without agglomeration of Poly(butyl acrylate) (PBA) and Poly(isobornyl acrylate) (PIBA) (Figure S1a,b, Supporting Information).^[^ ^25^ ^]^ Based on the results of the WAXS measurement, the Bragg formula can be used to calculate the layer spacing.^[^ ^37^ ^]^ The result ranges from 0.44 to 0.58 mm for the elastomers with *f* from 0 to 1 (Figure S1c, Supporting Information), indicating no aggregation in these copolymers. The excellent compatibility of the soft and hard segments also endows the elastomers with high optical transparency, which is above 90% when the sample is 1 mm thick (Figure 1b). By tuning the molar ratio of the IBA monomer, *f*, the mechanical properties of the resulting elastomers can be facilely adjusted in a wide range. As shown in Figure 1c, the elastomer with a relatively low IBA (*f* = 0.3) content is soft, weak, and ductile, exhibiting Young's modulus and fracture stress of 0.25 and 0.16 MPa, respectively. As the *f* is increased to 0.4, the stiffness and strength are simultaneously enhanced (Young's modulus and fracture stress of 0.94 and 2.04 MPa), without sacrificing the stretchability. Further increasing the content of IBA in the elastomer system (*f* = 0.5) results in a significant reinforcement of Young's modulus (14.7 MPa) and fracture stress (5.05 MPa), which comes at the expense of fracture strain. Compared with the elastomer without IBA (*f* = 0), the fracture stress and Young's modulus of the *f* = 0.5 elastomer are enhanced by 130‐ and 63‐fold, respectively (Figure 1d). The fracture toughness of the elastomers is also characterized using a trouser tearing test, and the result shows a similar trend to that of the tensile test (Figure S2, Supporting Information). The tearing energy is proportional to the added amount of IBA, indicating again that the introduction of a hard segment into the elastomer system benefits the mechanical performance. Besides high strength, high toughness, good softness, and excellent stretchability, the developed elastomers are also highly self‐recovering and possess a satisfactory self‐healing ability, which can rapidly restore the original mechanical properties upon loading–unloading cycles or partially heal the damage after damage (Figures S3 and S4, Supporting Information).

The remarkable mechanical properties of the elastomers are demonstrated in Figure 1e. It is shown that an elastomer (*f* = 0.4) is capable of withstanding a large deformation under stretch or knotted elongation. Although *f* = 0.5 elastomer shows the optimal mechanical performance, a fairly high Young's modulus is usually not desirable for adhesion.^[^ ^38^, ^39^ ^]^ We therefore select *f* = 0.4 elastomer as the model adhesive in the following sections due to its high energy dissipation ability yet low stiffness.

### 2.2 Adhesive Properties

**Figure 2:**
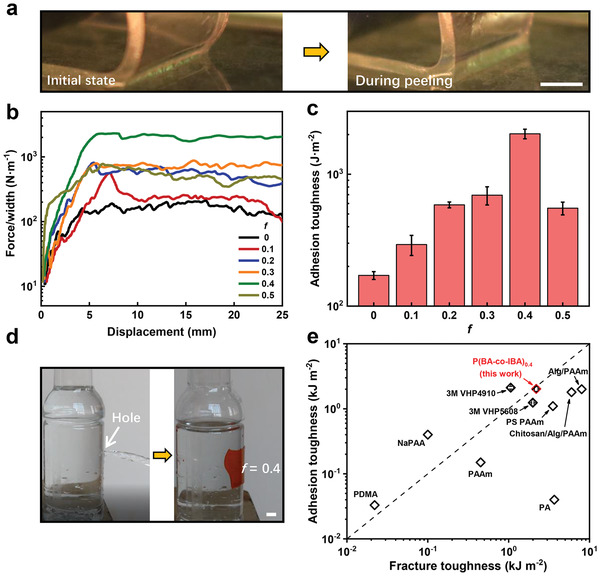
a) Photographs of the *f* = 0.4 elastomer during the 90° peeling test. The scale bar represents 5 mm. b) Force/width versus displacement curves of P(BA*‐co‐*IBA) elastomers with varied *f* during the 90° peeling test. Glass plate is used as the substrate. c) Corresponding adhesion toughness of P(BA*‐co‐*IBA) elastomers with varied *f*. d) The *f* = 0.4 elastomer acting as an instant sealant for a container with an obvious hole. The scale bar represents 10 mm. e) Comparison of adhesion and fracture toughness between the *f* = 0.4 elastomer in this work and other state‐of‐the‐art adhesives. PDMA is poly(dimethylacrylamide). NaPAA is sodium polyacrylate. PAAm is polyacrylamide. PS PAAm is phase‐separated polyacrylamide. PA is polyampholyte. Alg is alginate.

Next, we use a common testing method for adhesives, a 90° peeling test, to measure the adhesion properties of the P(BA‐*co*‐IBA) elastomers (Figure S5a, Supporting Information).^[^ ^34^, ^40^, ^41^ ^]^ Glass plate is utilized as a typical substrate for the measurement. Photographs of the *f* = 0.4 elastomer before and during peeling are manifested in **Figure** **2**a. The crack front of the elastomer becomes highly deformed as the measurement goes on to resist the interfacial separation, suggesting a tough adhesion of the elastomer to the substrate. Corresponding curves of force/width versus displacement of the elastomers with varied *f* are gathered in Figure 2b. It can be seen that the addition of IBA into the BA system gives rise to an obvious increase in the adhesion performance. As compared in Figure 2c, the neat PBA elastomer (*f* = 0) shows an adhesion toughness of 171 J m^−2^, which is an order of magnitude lower than that of the *f* = 0.4 elastomer (2026 J m^−2^). Although PBA is a common commercial adhesive, we consider that the relatively low energy dissipation capability limits it to achieving an extraordinarily high adhesion toughness. With the introduction of IBA into the PBA system, we observe an enhancement of the adhesion performance. As mentioned in the introduction part, the interfacial toughness of the elastomer‐solid bonding is related to two factors: intrinsic adhesion (interfacial interaction) and energy dissipation of the material around the interface. Because the adhesion between all elastomers and the substrate is physical (van der Waals interaction, hydrogen bonding, etc.), we attribute the dramatic enhancement in adhesion toughness to the intensified energy dissipation of the elastomer near the interface. Note that the *f* = 0.5 elastomer exhibits an abnormally low adhesion toughness although it exhibits the optimal mechanical properties. We consider that one possible reason is due to the rather high stiffness of the *f* = 0.5 elastomer (Young's modulus of which is orders of magnitude higher than other elastomers), which deteriorates its conformability for an effective contact.^[^ ^35^, ^38^, ^39^ ^]^ In other words, roughness always exists on the surface of a substrate. For the low‐modulus elastomer, almost full contact can be expected because it is able to deform and fill out the surface cavities, ensuring sufficient bonding sites that can transfer the stress for energy dissipation of the material near the interface. On the contrary, partial contact is expected when a high‐modulus elastomer is utilized,^[^ ^42^ ^]^ where the elastomer makes contact only close to the top of the highest asperities of the surface. In this case, binding sites are significantly decreased, hampering the efficient stress transfer for energy dissipation.

There are several influencing factors on the adhesion properties of the elastomer adhesives. Here, we use the *f* = 0.4 elastomer as an example and discuss the influencing factors on its adhesion performance. The first influencing factor is the specimen thickness. Because the energy dissipation of the material near the interface significantly depends on the size of the energy dissipation zone,^[^ ^43^ ^]^ a change in the thickness of the sample will affect the adhesion toughness.^[^ ^44^, ^45^ ^]^ As shown in Figure S6 in the Supporting Information, the elastomer shows a gradually increased adhesion toughness when the sample thickness is below 1 mm, indicating an insufficient energy dissipation during the interfacial separation. When the sample thickness is above 1 mm, the adhesion toughness achieves the plateau value, which means that the size of the energy dissipation zone of this material is around 1 mm.^[^ ^43^, ^44^ ^]^ The second factor is the testing rate,^[^ ^46^ ^]^ as the elastomers are viscoelastic (Figure S7, Supporting Information). In other words, the mechanical dissipation of the elastomers should be rate‐dependent, which, in turn, affects the adhesion performance. The evolution of force/width and corresponding adhesion toughness of the *f* = 0.4 elastomer with different thicknesses under a variety of testing velocities is shown in Figure S8 in the Supporting Information. For all samples, despite possessing distinct sample thickness, the adhesion toughness gradually increases with the rise of testing velocity, due to the improvement of energy dissipation capability of the elastomer. The 0.4 mm thick sample exhibits a systematical lower adhesion toughness compared with the 1 and 3 mm samples at any testing velocity, which is consistent with the previous results and hypothesis indicating that the elastomer should possess a thickness of at least 1 mm to maximize the size of energy dissipation zone. Also, note that the elastomer tested at a velocity of 200 mm min^−1^ shows a similar adhesion toughness to that of the one tested at 100 mm min^−1^. This could be explained as follows. The elastomer working at a relatively high rate possesses an improved energy dissipation capability and high Young's modulus, a high dissipation benefits the adhesion while a high stiffness deteriorates the contact.^[^ ^38^, ^39^ ^]^ Such a tradeoff gives rise to the plateau‐like adhesion toughness of the material at a high testing rate. We also examine the effect of sample width on the adhesion performance of the *f* = 0.4 elastomer. The results show that changing sample width does not influence the adhesion toughness during the 90° peeling test (Figure S9, Supporting Information). Increasing the sample width can elevate the peeling force. However, the adhesion toughness is calculated by the peeling force over sample width, which should be a normalized value. A size‐dependent effect in the width direction is not likely to be observed as that in the thickness direction.

As a comparative study, we also employ the lap shear test to measure the adhesion properties of the elastomer adhesives (Figure S5b, Supporting Information).^[^ ^45^, ^47^ ^]^ During the test, all samples show an interfacial failure once the displacement is large enough, without breaking either the elastomers or the substrate. As shown in Figure S10a in the Supporting Information, adding IBA into the PBA system significantly reinforces the adhesion strength. The *f* = 0.5 elastomer shows the optimal adhesion strength of 877 kPa, which is 22‐fold that of neat PBA (39 kPa). The corresponding energy release rate is calculated from the results and is shown in Figure S10b in the Supporting Information, which exhibits a similar trend to that of the 90° peeling test. The energy release rate increases gradually as *f* is increased from 0 to 0.4, which shows a clear drop when *f* reaches 0.5. Because the substrate is the glass plate, which can be deemed as extremely stiff and does not deform. That is, the calculation of the energy release rate depends heavily on Young's modulus of the adhesive.^[^ ^45^, ^48^ ^]^ The significantly increased stiffness of the *f* = 0.5 elastomer leads to the sharply reduced energy release rate, and the synergy of effective contact (low Young's modulus) and massive energy dissipation near the interface enables the *f* = 0.4 to demonstrate the optimal adhesion performance. Note that the energy release rate measured by the lap shear test differs from that determined by the 90° peeling test. We consider that there are two potential reasons. On the one hand, sample geometry such as volume is not the same for the two adhesion tests. On the other hand, the testing velocities are different, which has significant impact because the elastomer is highly viscoelastic. The strong adhesion of the *f* = 0.4 elastomer is also durable. Using a stress relaxation test, we compare the evolution of adhesion strength with time for the *f* = 0.4 and *f* = 0 elastomers at a fixed strain of 10%, respectively. The *f* = 0.4 elastomer maintains a high and stable adhesion performance with the increase of time, superior to that of the *f* = 0 elastomer (Figure S11, Supporting Information).

By virtue of the excellent adhesion performance of the elastomer, we can employ it in our daily life as an instant, efficient, and durable adhesive. As a demonstration, we first exploit this elastomer as an instant sealant for damaged goods. Figure 2d shows that the *f* = 0.4 elastomer can easily prevent water leakage of a container with a macroscopic hole. The durable adhesion also allows this elastomer to be used as a long‐lasting adhesive, which can stably adhere a mobile phone to a flat surface for days (Figure S12a, Supporting Information). Similarly, we can replace the adhesive layer of a conventional hook with the elastomer, which works perfectly in the air for 120 h (Figure S12b, Supporting Information). On the contrary, the hook using the *f* = 0 elastomer as the adhesive layer drops down only after 15 min (Figure S12b, Supporting Information).

To further highlight the superb adhesion and mechanical performance of the viscoelastic elastomers, we make a systematic comparison between the elastomer adhesives developed in this work and the state‐of‐the‐art adhesives in industrial and biomedical fields in Figure 2e. The adhesion energy is plotted as a function of the fracture energy of the material. Compared with two types of 3 M tapes that are usually used as commercial adhesives, the *f* = 0.4 elastomer shows a clear advantage in terms of fracture toughness or adhesion toughness. On the other hand, the elastomer also exhibits superior fracture and adhesion toughness to most of the common hydrogel adhesives that are utilized in biomedical fields.^[^ ^49^ ^]^ Only some hydrogels based on alginate or chitosan demonstrate close adhesion toughness to the elastomer while showing improved fracture properties.^[^ ^50^ ^]^ Note that hydrogel adhesives are generally water‐rich and lose their functions once dehydrated, which is not an issue for elastomer adhesives.^[^ ^51^ ^]^

### 2.3 Instantaneity, Repeatability, and Stability of the Tough Adhesion

**Figure 3:**
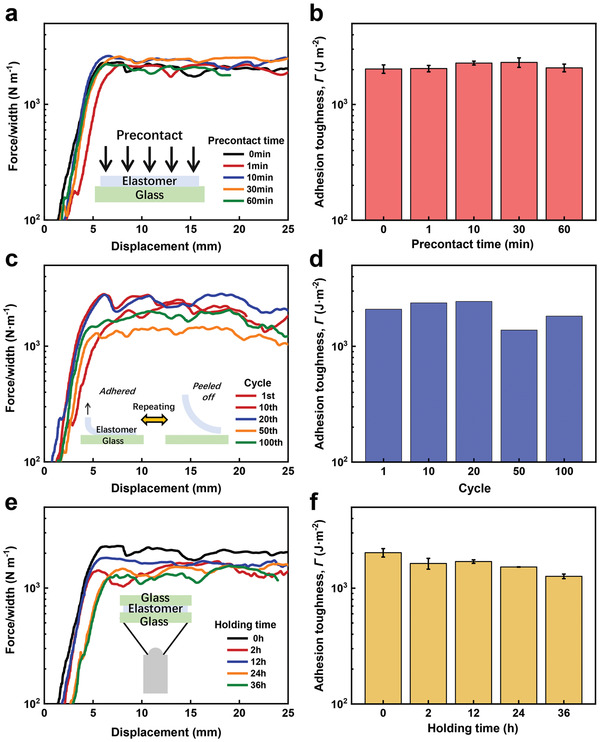
a) Force/width versus displacement curves of the *f* = 0.4 elastomer that adheres to glass under different precontact times during the 90° peeling test. b) Adhesion toughness of the *f* = 0.4 elastomer under different precontact times. c) Force/width versus displacement curves of the *f* = 0.4 elastomer at different adhesion cycles. d) Adhesion toughness of the *f* = 0.4 elastomer at different adhesion cycles. e) Force/width versus displacement curves of the *f* = 0.4 elastomer that holds a weight of 500 g for different times. f) Adhesion toughness of the *f* = 0.4 elastomer with different holding times.

The tough adhesion of the elastomers to substrates is also instant, repeatable, and stable. Such merits are demonstrated here using the *f* = 0.4 elastomer via the 90° peeling test. The instantaneity of the tough adhesion is first studied. Before measuring the adhesion toughness, the elastomer is attached to a glass plate under the same pressure of 1 N for different precontact times. **Figure** **3**a shows the precontact time‐independent adhesion properties of the elastomer, which shows constant force/width–displacement curves no matter the contact time is short or long. The resulting adhesion toughness maintains a high level, which achieves as high as 2000 J m^−2^ (Figure 3b). Similar results are obtained for the *f* = 0.5 elastomer although it shows a relatively low adhesion toughness (Figure S13, Supporting Information).

Besides the instantaneity, the adhesion is repeatable. Repeated 90° peeling tests on the same *f* = 0.4 elastomer show almost unchanged force/width–displacement curves and corresponding adhesion toughness (Figure 3c,d and Figure S14, Supporting Information), suggesting the satisfactory repeatability of the tough adhesion. This result should be attributed to two reasons. First, the adhesion between the elastomer and the substrate is physical (Van der Waals interaction, hydrogen bonding, etc.). Second, the elastomer can rapidly self‐recover once it is unloaded. Loading–unloading tests show that the *f* = 0.4 elastomer is able to immediately restore the hysteresis (Figure S3b, Supporting Information). That is, this physical elastomer can quickly recover the internal damage once unloaded, allowing satisfactory reusability.

Finally, the durability of the tough adhesion is also investigated. We connect two glass plates using the *f* = 0.4 elastomer, the bottom of which holds a weight of 500 g. The total setup is then soaked in water. After a certain amount of time, the adhesion toughness between the elastomer and the upper glass plate is examined. Figure 3e gathers the force/width–displacement curves of the elastomer–substrate adhesion with different holding times. Corresponding adhesion toughness justifies the stability of the adhesion, which slightly decreases even the holding time reaches 36 h (Figure 3f).

The results in this section verify that the tough adhesion of the elastomer to substrates is simultaneously instant, repeatable, and stable, which is promising for a variety of practical applications.

### 2.4 Adhesion Mechanism

The results in the former sections indicate that the viscoelastic elastomer in this work is highly adhesive, and the hypothesis attributes this to a synergy of high intrinsic work of adhesion and massive energy dissipation around the interface. Therefore, in this section, we attempt to verify the assumption by examining the influence of these two factors.

**Figure 4:**
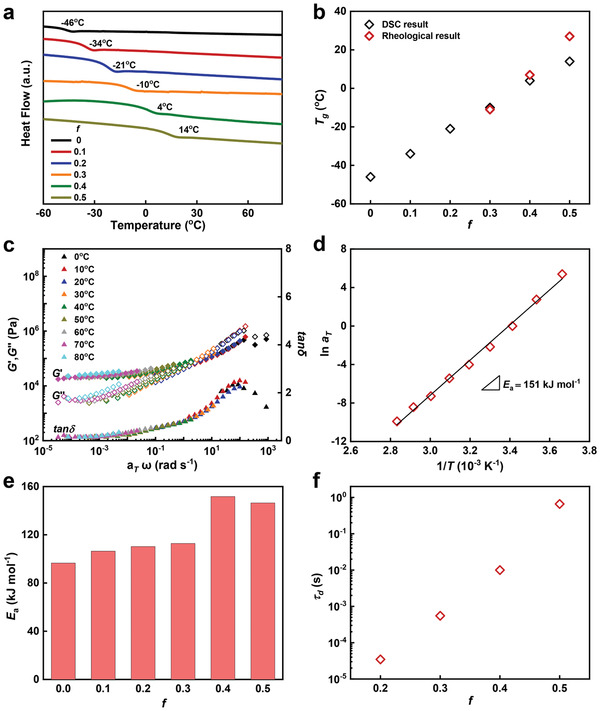
a) Differential scanning calorimetry (DSC) curves of P(BA*‐co‐*IBA)*_f_* elastomers with varied *f*. b) Glass transition temperatures (*T*_g_) of P(BA*‐co‐*IBA) elastomers with varied *f* obtained by DSC and rheological test, respectively. c) Typical master curves of *G*', *G*", and tan*δ* of the *f* = 0.4 elastomer with 20 °C as the reference temperature. d) Arrhenius plot for the temperature‐dependent shift factors (*a*_T_). The apparent activation energy (*E*_a_) is calculated from the slope of the curve. e) Calculated *E*_a_ for the P(BA‐*co*‐IBA) elastomers with varied *f*. f) The relaxation time (*τ*_d_) of the P(BA‐*co*‐IBA) elastomers with varied *f*.

The intrinsic work of adhesion generally relies on the bonding properties between the adhesive and the substrate.^[^ ^21^ ^]^ For physical contact (Van der Waals interactions, hydrogen bonding, etc.) in this work, the mobility of polymer chains plays a significant role in determining this factor. First, the glass transition temperature (*T*_g_) of the elastomers in this work is measured, which greatly affects the chain mobility of polymers.^[^ ^25^ ^]^ Differential scanning calorimetry (DSC) and rheological tests are both carried out, and the results are shown in **Figure** **4**a and Figure S15 in the Supporting Information. All elastomers possess a *T*_g_ that is lower than room temperature, which increases linearly with the augment of the IBA content (Figure 4b). That is, although the introduction of IBA decreases the chain mobility of the resulting elastomer, the polymer chains of all elastomers are still quite flexible due to the low *T*_g_.

For further confirmation, we investigate apparent activation energy (*E*_a_) and the longest relaxation time of the polymer chains using rheological tests. Frequency sweeps of elastomers at a shear strain of 0.1% at different temperatures are carried out. Master curves of storage modulus (*G*'), loss modulus (*G*"), and loss factor (tan*δ*) are obtained by time–temperature superposition shifts (TTS) at the 20 °C reference temperature. The corresponding result of the *f* = 0.4 elastomer is exhibited as an example in Figure 4c. Master curves for other elastomers with varied *f* are summarized in Figure S16a in the Supporting Information. All elastomers show a frequency‐dependent storage and loss modulus, demonstrating obvious viscoelasticity. The molar ratio of the hard segment (IBA) has a significant influence on the characteristic relaxation time (*τ*_d_) and apparent activation energy (*E*_a_). The *E*_a_ is obtained from the slope of the Arrhenius plots for the shift factor, *a*_T_ (Figure 4d and Figure S16b, Supporting Information).^[^ ^25^, ^52^ ^]^ As summarized in Figure 4e, the *E*_a_ of the elastomers gradually increases with the elevation of the IBA content, indicating an enhanced bonding between molecular chains and attenuated chain mobility. According to the spectrum of tan*δ*, we can also figure out the *τ*_d_ of the elastomers.^[^ ^52^ ^]^ As manifested in Figure 4f, the *τ*_d_ at 293 K of the elastomer rises with *f*, increasing from 10^−5^ (*f* = 0.2) to 10^−1^ s (*f* = 0.5). Note that all these elastomers have a rather short relaxation time even the gap is on several orders of magnitude. Such results are consistent with the *T*_g_ results and suggest that although the introduction of IBA into the PBA system deteriorates the chain mobility, the polymer chains of these elastomers are still quite mobile, which endows the elastomer with a desirable intrinsic work of adhesion.

**Figure 5:**
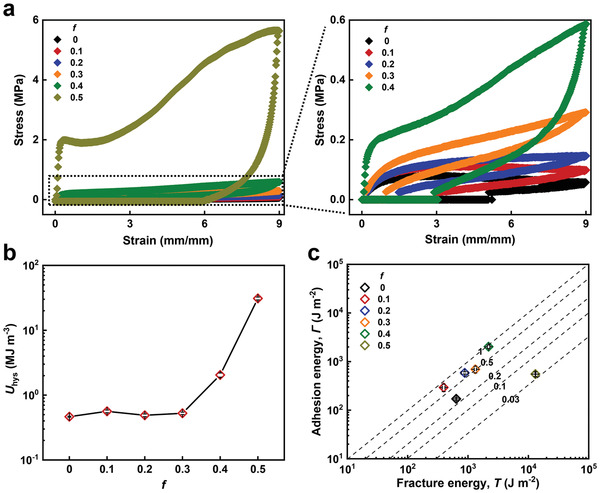
a) Tensile loading–unloading stress–strain curves of the P(BA*‐co‐*IBA) elastomers with varied *f* at a fixed strain of 900%. b) Corresponding hysteresis (*U*_hys_) of elastomers with varied *f* calculated from the loading–unloading curves. c) Adhesion energy (*Γ*) versus fracture energy (*T*) for the P(BA*‐co‐*IBA) elastomers with varied *f*.

Another determinant of adhesion properties is the energy dissipation of the material around the elastomer–substrate interface.^[^ ^21^ ^]^ Therefore, we compare the energy dissipation capability of the elastomers with varied *f*. Different amount of IBA is introduced into BA through copolymerization to enhance the energy dissipation capability. A loading–unloading test at a fixed strain can reflect the amount of energy dissipation during the deformation of the sample. As shown in **Figure** **5**a, the introduction of IBA leads to a significant improvement in energy dissipation during a loading–unloading cycle. The hysteresis of the neat BA at a strain of 900% is ≈0.47 MJ m^−3^, while that of the *f* = 0.5 elastomer is several orders of magnitude higher (30.97 MJ m^−3^). The hysteresis (*U*_hys_) of the elastomers increases slowly when *f* is below 0.4, resulting in a slight increase of adhesion toughness in these elastomers. When *f* is increased to 0.4, the hysteresis is enhanced dramatically (Figure 5b), giving rise to a significant increase in adhesion toughness. Note that the elastomer with *f* = 0.5 possesses the optimized energy dissipation capability. However, the adhesion toughness of this elastomer is relatively low. We plot the adhesion toughness (*Γ*) versus the fracture toughness (*T*) for P(BA*‐co‐*IBA) elastomers at varied *f*, and the slope (*Γ*/*T*) of which indicates the effective contribution of energy dissipation to adhesion energy during interfacial separation (Figure 5c). It can be seen that the elastomers with a low *f* demonstrate a slope in the range from 0.2 to nearly 1 (Figure 5d). The *f* = 0.4 elastomer shows the optimal efficiency approaching 1, indicating a significant contribution of energy dissipation to the adhesion toughness. In contrast, the efficiency for the *f* = 0.5 elastomer is merely above 0.03, which means that an extremely limited energy dissipation of this elastomer is exploited for the adhesion performance although it is highly energy dissipative.

### 2.5 Universality

**Figure 6:**
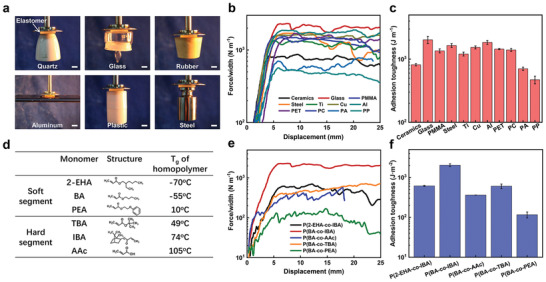
a) The universal adhesion of the *f* = 0.4 elastomer to a variety of substrates. The scale bars represent 10 mm. b) Force/width versus displacement curves during the 90° peeling test for the *f* = 0.4 elastomer that is adhered to different substrates. c) Calculated adhesion toughness of the *f* = 0.4 elastomer to various substrates. PMMA is polymethylmethacrylate, PET is polyethylene terephthalate. PC is polycarbonate, PA is polyamide, PP is polypropylene. d) Abbreviations and chemical structures of various acrylate monomers that can be used to fabricate tough adhesives. The *T*_g_ of their corresponding homopolymers are given, based on which we can divide them into two categories, a soft segment and a hard segment. e) Force/width versus displacement curves during the 90° peeling test of various P(A*‐co‐*B) elastomers that are adhered to the glass. “A” represents the soft segment and “B” represents the hard segment. The *f* of the hard segment is fixed as 0.4. f) Calculated adhesion toughness of various elastomers that are adhered to the glass.

Since the interaction between the elastomer and the substrate is based on physical interactions, the adhesion is universal. The high mobility of the *f* = 0.4 elastomer chains enables it to adhere to a variety of solid substrates, including, but not limited to quartz, glass, rubber, aluminum, plastic, and steel (**Figure** **6**a). The 90° peeling test indicates that the elastomer shows a high adhesion toughness when it is attached to different surfaces (Figure 6b). The adhesion toughness ranges from 472 to 2026 J m^−2^ (Figure 6c).

On the other hand, the preparation formula of elastomer adhesives is universal as well. By distinguishing the *T*_g_ of the corresponding homopolymer, we can determine the monomer to constitute either the soft or the hard segment of the resulting elastomer. A variety of elastomer adhesives with high adhesion toughness can be fabricated by combining a soft and a hard segment with contrasting *T*_g_ (Figure 6d). Using the glass plate as a typical substrate, 90° peeling tests show that all these elastomers with *f* = 0.4 exhibit a desirable adhesion toughness above 100 J m^−2^ (Figure 6e,f).

### 2.6 Soft Electronics Enabled by the Tough Adhesion

**Figure 7:**
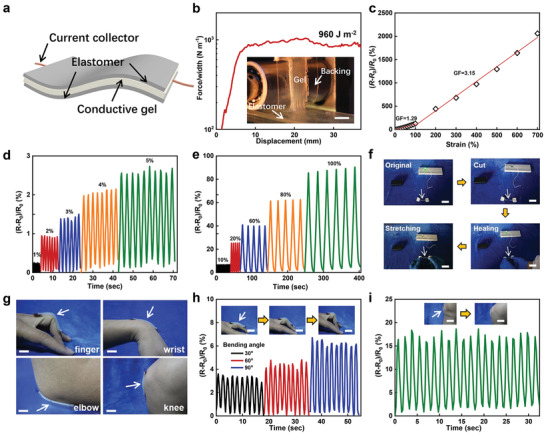
a) Illustration of the multilayer sensor consisting of two pieces of *f* = 0.4 elastomer and a piece of a conductive hydrogel. b) Force/width–displacement curve during the 90° peeling test of the hydrogel that is adhered to the elastomer. Corresponding adhesion toughness achieves 960 J m^−2^. The scale bar represents 10 mm. c) The resistance variation of the sensor as a function of strain. The GF is determined by the slope of the curve. d) Electrical performance of the sensor under small strains. e) Electrical performance of the sensor under large strains. f) Photographs showing that the sensor is able to self‐heal after cut and restore the function even under stretch. Scale bars represent 10 mm. g) Conformable adhesion of the *f* = 0.4 elastomer to different body parts. Scale bars represent 20 mm. h) Finger movement at different angles monitored by the multilayer sensor. Scale bars represent 20 mm. i) Body movement monitored by the multilayer sensor that is adhered to the knee. Scale bars represent 20 mm.

Depending on the remarkable adhesion performance of the elastomer adhesives on various surfaces, numerous applications can be expected. As an example, here we demonstrate a multilayer soft sensor using the *f* = 0.4 elastomer as the adhesive and protective layer simultaneously because conductive hydrogels are usually not adhesive to skins and dehydrate easily in air. Encapsulating hydrogels with a hydrophobic frame has been proven highly effective to prevent water evaporation.^[^ ^31^, ^53^ ^]^ **Figure** **7**a shows the structure of the multilayer sensor. A salt‐containing hydrogel connected by two copper wires is sandwiched in two layers of the *f* = 0.4 elastomer, playing the role of body movement monitoring. Because the gel is in situ polymerized between two pieces of the *f* = 0.4 elastomer using benzophenone (BP) as initiator, the gel–elastomer intrinsic adhesion is originated from chemical grafting as reported in previous work.^[^ ^31^, ^54^ ^]^ The interfacial toughness between the hydrogel and the elastomer layers is first investigated by the 90° peeling test. Significant deformation of the hydrogel is observed when it is peeled off from the elastomer (Figure 7b). Corresponding adhesion toughness achieves 960 J m^−2^. Another simple demonstration to visualize the tough adhesion between the elastomer and the hydrogel is shown in Figure S17 in the Supporting Information. When the elastomer is elongated, the adhered hydrogel also deforms significantly. Once recovered to the initial strain, both materials retreat to the original state and no delamination between the two layers is observed. Directly attaching the elastomer to soft surfaces can also lead to tough adhesion. Figure S18a in the Supporting Information shows the optical image of a piece of pigskin closely connecting to the *f* = 0.4 elastomer. The corresponding force/width–displacement curve indicates that the adhesion toughness approaches 100 J m^−2^ (Figure S18b, Supporting Information).

The tough adhesion between the elastomer and skins ensures the proper function of the resulting multilayer sensor with a wide monitoring window. As shown in Figure 7c, in the small deformation region at a strain of 0–100%, the gauge factor (GF) of the device is 1.29, which increases to 3.15 when the deformation becomes large (a strain of 100–700%). In both regions, the GF shows a good linear relationship with the strain of the device. The results of resistance variation of the sensor under different strain cycles show that the device can distinguish inconspicuous deformations of strain at 1–5% and 10–100% (Figure 7d,e). Note that the sensor does not immediately restore the original resistance value once the strain becomes high, i.e., residual strain exists. This is due to the viscoelasticity of the elastomer layer, which recovers relatively slowly once being stretched (full recovery within 3 min, as shown in Figure S3, Supporting Information). In this case, it is better to match the elastomer with applications that operate at specific temperature or frequency, under which the elastomer can show an elastic behavior. By virtue of the good self‐healing ability of the elastomer and the tough elastomer–gel bonding, the sensor can restore the conductivity once cut. The light‐emitting diode light remains on when the cut sensor is self‐healed and afterward stretched to a certain length (Figure 7f).

Next, we demonstrate the body movement monitoring of the hybrid sensor due to its good adhesion to skins.^[^ ^55^, ^56^ ^]^ As shown in Figure 7g, the sensor can confirm and deform synchronously with different body parts. Using finger movement as an example, we show that the sensor can precisely detect the bending motion with different angles (30°, 60°, and 90°) and convert the motions into electric signals (Figure 7h). We also demonstrate the noiseless and stable output of signals when using the sensor to monitor the movement of the knee during walking (Figure 7i).

## 3 Conclusion

In summary, we report the simple fabrication of a transparent and self‐healing elastomer that shows instant, tough, yet repeatable adhesion to various soft and hard surfaces. The strategy is to tune the glass transition temperature of the elastomer by using two acrylate monomers as the soft and hard segments, respectively. The resulting elastomer, with a *T*_g_ slightly lower than room temperature, shows a short relaxation time and high energy dissipation capability, which enable high mobility of polymer chains and massive mechanical dissipation near the interface during interfacial separation. As a result, tough yet repeatable adhesion of the elastomer is achieved. Numerous acrylate monomers are applicable to develop such efficient adhesives and the adhesion is universal to a variety of soft and hard surfaces, opening the opportunity of utilizing the elastomers for detachable devices such as sensors, monitors. We hope this work provides new insight into the development of physical adhesives, which is of significance in terms of cost reduction and recycling of industrial devices.

## 4 Experimental Section

### Materials

Ethyl acrylate (EA), n‐butyl acrylate (BA), 2‐ethylhexyl acrylate (2‐EHA), acrylic acid (AAc), isobornyl acrylate (IBA), *tert*‐butyl acrylate (TBA), ethylene glycol phenyl ether acrylate (PEA), *N*,*N*′‐methylenebis (acrylamide) (MBA, 99%), sodium chloride (NaCl) were purchased from Aladdin corporation. 2‐Hydroxy‐4′‐(2‐hydroxyethoxy)‐2‐methylpropiophenone (Irgacure 2959) and benzophenone (BP) were provided by TCI corporation. Bovine serum albumin (BSA, 98%), acrylamide (AAm, 98%) were purchased from Sigma‐Aldrich Inc. All chemicals were used as received.

### Preparation of the Elastomer Adhesives

The desired amount of two liquid monomers (“A” with a *T*_g_ of its homopolymer below the room temperature, “B” with a *T*_g_ of its homopolymer above the room temperature) were mixed with the initiator Irgacure 2959 (photoinitiator, 0.1 mol% of monomers), which were stirred evenly and injected into a closed reaction cell consisting of two glass plates separated by a 1 mm silicon spacer. Copolymers were synthesized via free radical polymerization initiated by ultraviolet light (intensity of 50 W and wavelength of 365 nm) for 2 h. The as‐prepared elastomer was denoted as P(A‐*co*‐B)*_f_*, where *f* represents the molar ratio of the “B” monomer and is calculated as *f* = *M*_B_/(*M*_A_+*M*_B_).

### Fabrication of the Multilayer Sensor

BSA/PAAM DN network hydrogel was in situ polymerized between two pieces of *f* = 0.4 P(BA‐*co*‐IBA) elastomers, which worked as a conductive layer in the sandwich devices. First, monomers, BSA (300 mg mL^−1^) and AAM (20 wt%), crosslinker, MBA (0.03 mol% of AAm), initiator, BP (6 mg mL^−1^), and salt NaCl (0.5 m) were added into 5 mL of deionized water and stirred evenly. Oxygen was removed by nitrogen pumping. The precursor solution was then added into a mold using two pieces of *f* = 0.4 P(BA‐*co*‐IBA) elastomer on each inside. The BSA network was formed in a water bath at 80 °C for 10 min. Afterward, the polymerization of the PAAM network was initiated by BP under UV light (365 nm wavelength, 50 W) for 2 h. As a result, a sandwiched sensor with the elastomer as the adhesive layer was fabricated.

### WAXS and SAXS

The WAXS and SAXS measurements of as‐prepared P(BA‐*co*‐IBA) elastomers (thickness of 1 mm) were performed on a modified Xeuss system of Xenocs France equipped with a semiconductor detector (Pilatus 100 K, DECTRIS, Swiss) attached to a multilayer focused Cu K*α* X‐ray source (GeniX3D Cu ULD, Xenocs SA, France), generated at 50 kV and 0.6 mA. The wavelength (*λ*) of the X‐ray radiation was 0.154 nm. The Bragg d‐spacing was calculated as *d* = *λ*/2sin*θ*,^[^ ^57^ ^]^ where 2*θ* was the scattering angle.

### Differential Scanning Calorimetry

The DSC measurement was performed in a heat‐cool cycle (25 °C jump to 125 °C, 10 °C min^−1^; isothermal 125 °C, 10 min; 125 to −100 °C, 10 °C min^−1^; −100 °C jump to 125 °C, 10 °C min^−1^), wherein the thermal transitions for the last heating cycle were recorded. The glass transition temperature (*T*_g_) was determined by the inflection point of the heat capacity with temperature sweep.

### Optical Microscope

To investigate the self‐recovery ability of the elastomer, the evolution of a cut on an elastomer with a SMART‐POL polarizing microscope was observed. Photographs of the cut and healed elastomer were taken using a Canon EOS 7500 camera.

### Mechanical Test

Mechanical properties reflected by uniaxial tensile, load–unloading, cyclic loading–unloading, trouser‐tearing, lap‐shear, and 90° peeling tests, were measured by a WSM‐10 kN tensile machine.

### Tensile Test

Dumbbell‐shaped samples (50 mm in length, 4 mm in width, and 1 mm in thickness) were used for the uniaxial tensile test at a testing velocity of 100 mm min^−1^ in air. The integral area of the obtained stress–strain curves was defined as the work of extension at fracture. For the loading–unloading test, samples were stretched to a certain strain, followed by unloading at the same velocity (varied from 10 to 200 mm min^−1^). The waiting time for the recovery process was varied from 0 to 10 min. For the cyclic loading–unloading test, the samples were stretched to a strain, followed by unloading at the same strain rate, and the sequential loading–unloading cycles with increased stretch ratios were performed without waiting time. For the trouser‐tearing test, samples (length 50 mm, width 8 mm, thickness 1 mm) with an initial notch of 20 mm in the middle along the length direction were used. The two legs of the sample were clapped and displaced at a fixed velocity of 50 mm min^−1^. Tearing energy was calculated as *T* = 2*F*/*t*,^[^ ^44^ ^]^ where *F* is the steady‐state tearing force and *t* is the sample thickness.

### Adhesion Test

For the 90° peeling test, the backside of the sample was glued to the polyethylene terephthalate (PET) film to prevent elongation. All samples had a sufficiently long length, the width was changed from 8 to 15 mm and the thickness was changed from 0.2 to 3 mm. For most of the tests, samples had a geometry of 30 mm in length, 10 mm in width, and 1 mm in thickness. The testing velocity was 50 mm min^−1^. The adhesion toughness was defined as the average load per width, *F*/*w*,^[^ ^40^, ^58^ ^]^ where *F* is the steady‐state peeling force and *w* is the sample width. For the lap‐shear test, the sample was cut into a 20 × 20 mm^2^ square with a thickness of 10 mm, and the testing velocity was 10 mm min^−1^. The adhesion strength (*τ*_s_) was defined as the maximum tensile force (*F*_max_) per nominal contact area as *τ*_s_ = *F*_max_/*wl*,^[^ ^58^, ^59^ ^]^ where *w* and *l* are the width and length of the contact area, respectively. The corresponding energy release rate was calculated as *G* = (*F/w*)^2^/(4*Eh*),^[^ ^47^ ^]^ where *E*, *h*, and *w* are the elastic modulus, thickness, and width of elastomers, respectively. For the relaxation test, a fixed strain of 10% was applied to the lap shear setup with an elastomer as the adhesive. The evolution of the adhesion force and strength was recorded as a function of time.

### Rheological Test

Samples were cut into a disk shape (thickness 1 mm, diameter 25 mm) and the rheological tests were performed on an MCR 302 rheometer. The temperature sweep was performed at a shear strain of 0.1% and a frequency of 1 rad s^−1^. For the frequency sweep at different temperatures, the test was conducted in a frequency range of 0.1–100 rad s^−1^ at a shear strain of 0.1%. According to the principle of time–temperature superposition (TTS), master curves of storage modulus *G*’, loss modulus *G*”, and loss factor tan*δ* were constructed using a reference temperature of 20 °C. According to the Arrhenius temperature‐dependent shift factor diagram, the apparent activation energy *E*_a_ was calculated from the slope of the plot of ln*a*_T_ versus 1/*T* (*a*_T_ = *A*e*^E^* ^a^ *^/RT^*),^[^ ^52^ ^]^ where *a*_T_, *R*, *A* are the shift factor, molar gas constant, and pre‐exponential factor, respectively.

### Conductivity Measurement

The electrochemical performance of the resistive sensor was calculated using a Fluke 3000FC electrochemical workstation. The resistance change rate of the resistive sensor was calculated as Δ*R*/*R*_0_ = (*R* − *R*_0_)/*R*_0_*100%,^[^ ^60^ ^]^ where *R*_0_ is the resistance of the original sample and *R* is the real‐time resistance value. The GF was calculated from the slope of the curve of the resistance change rate (Δ*R*/*R*_0_) versus strain.

### Informed Consent

For the experiments including human research participants (experiments with sensors), informed consent was obtained from all participants prior to the research.

### Statistical Analysis

All experiments were conducted with a minimum of *N* = 5 for each data point; and the data obtained were expressed as the mean standard deviation. The error bars in all figures indicated the standard errors.

## Conflict of Interest

The authors declare no conflict of interest.

## Supporting information

Supporting Information

## Data Availability

The data that support the findings of this study are available from the corresponding author upon reasonable request.
