# Supplementary material for: Tough, Instant, and Repeatable Adhesion of Self‐Healable Elastomers to Diverse Soft and Hard Surfaces
Source: Adv Sci (Weinh). 2022 Feb 20;9(12):2105742. doi: 10.1002/advs.202105742 (PMC9036032; doi:10.1002/advs.202105742)
Supplement: Supplementary file 1 — Supporting Information [file ADVS-9-2105742-s001.pdf]

ADVANCED SCIENCE
Open Access

Supporting Information

for *Adv. Sci.*, DOI 10.1002/advs.202105742

Tough, Instant, and Repeatable Adhesion of Self-Healable Elastomers to Diverse Soft and Hard Surfaces

Ke Li, Xingjie Zan, Chen Tang, Zhuangzhuang Liu, Jianghuan Fan, Gang Qin, Jia Yang, Wei Cui, Lin Zhu and Qiang Chen**

Supporting Information

Tough, Instant, and Repeatable Adhesion of Self-Healable Elastomers to Diverse Soft and Hard Surfaces

Ke Li [1, 3, #], Xingjie Zan[1, 2, #], Chen Tang [1, 3], Zhuangzhuang Liu [1], Jianghuan Fan [1], Gang Qin [3], Jia Yang [3], Wei Cui[4, *], Lin Zhu [2], Qiang Chen [1, 2, 3, *]

[1] Wenzhou Institute, University of Chinese Academy of Sciences, Wenzhou, China, 352001.

[2] Oujiang Laboratory (Zhejiang Lab for Regenerative Medicine, Vision and Brain Health), Wenzhou, Zhejiang, China, 325000.

[3]School of Materials Science and Engineering, Henan Polytechnic University, Jiaozuo, China, 454000.

[4]College of Polymer Science and Engineering, State Key Laboratory of Polymer Materials Engineering, Sichuan University, Chengdu, China, 610065.

[#] Equal contribution.

* Corresponding author: Wei Cui, cuiwei@sci.hokudai.ac.jp

Qiang Chen, chenqiang@ucas.ac.cn

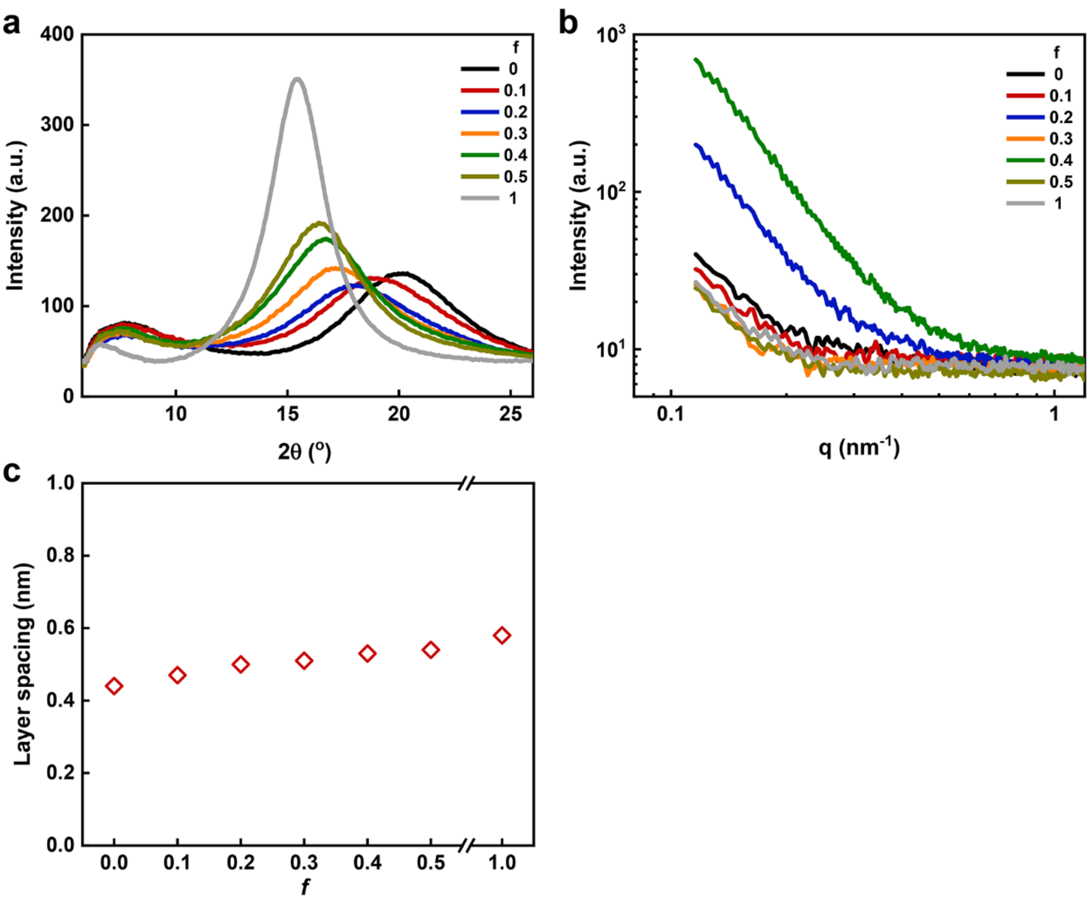

Figure S1. a) Wide-angle X-ray diffraction scattering (WAXS) measurements of P(BA-co-IBA) elastomers with varied *f*. b) Small-angle X-ray diffraction scattering (SAXS) measurements of P(BA-co-IBA) elastomers with varied *f*. c) The layer spacing for the elastomers with varied *f* based on the Bragg formula using the results of the WAXS measurement.

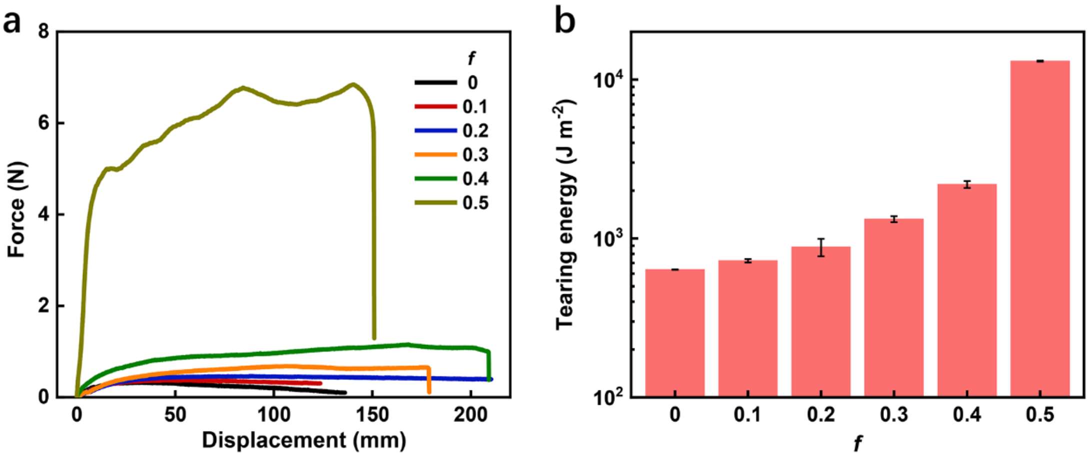

Figure S2. a) Force-displacement curves of the P(BA-co-IBA) elastomers with varied *f* during the trouser tearing tests. b) Calculated tearing energy of the P(BA-co-IBA) elastomers with varied *f*.

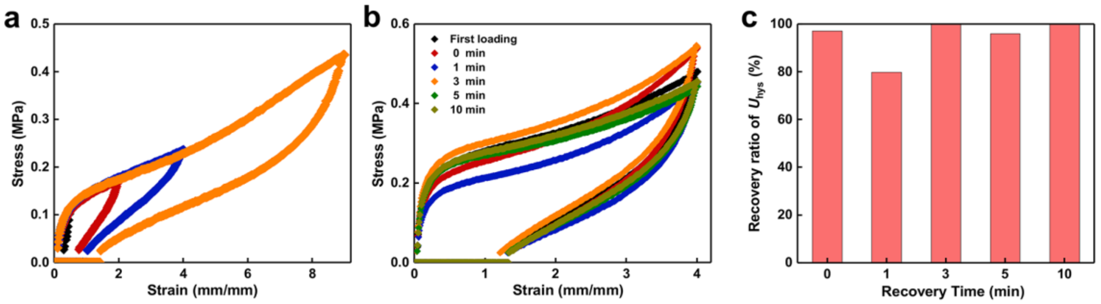

Figure S3. a) Successive cyclic load-unloading stress-strain curves of the *f* = 0.4 elastomer at different strains. b) Load-unloading stress-strain curves of the *f* = 0.4 elastomer with different resting times at a fixed strain of 400%. c) Recovery ratio defined by comparing the hysteresis (U_{hys}) of the *f* = 0.4 elastomer with different resting times with the hysteresis of the original sample.

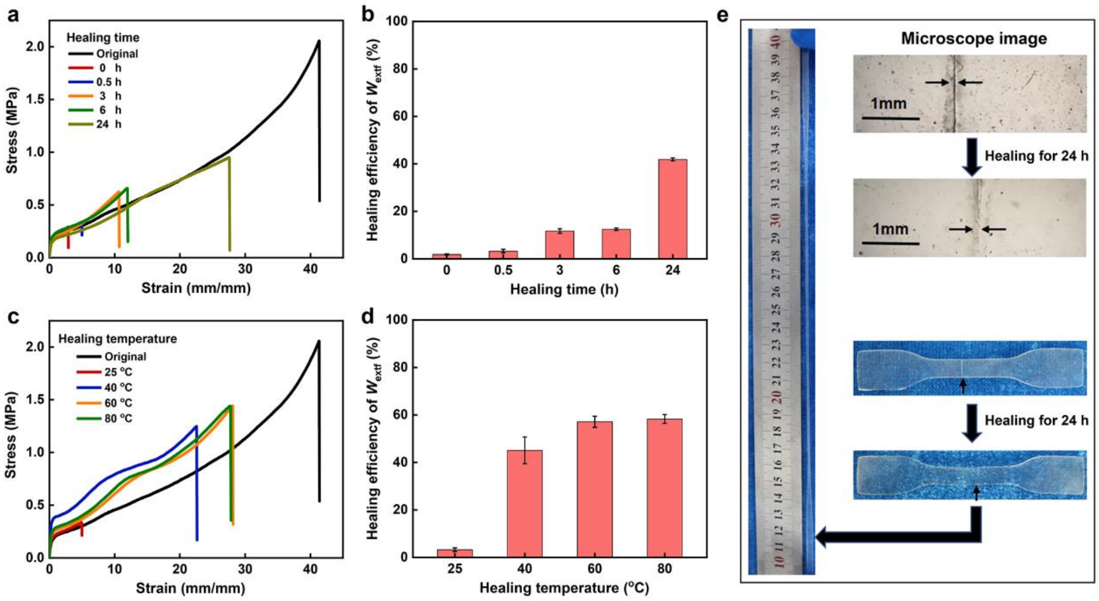

Figure S4. a) Stress-strain curves of the original and cut *f* = 0.4 elastomer with different healing times at 25 °C. b) Healing efficiency defined by comparing the work of extension at fracture (W_{extf}) of the *f* = 0.4 elastomer with different healing times with the hysteresis of the original sample. c) Stress-strain curves of the original and cut *f* = 0.4 elastomer with different healing temperatures for 0.5 h. d) Healing efficiency defined by comparing the work of extension at fracture (W_{extf}) of the *f* = 0.4 elastomer with different healing temperatures with the hysteresis of the original sample. e) Optical images and photographs showing the healing process of the *f* = 0.4 elastomer in 24 hours. The healed sample is still highly stretchable.

Self-recovery and self-healing properties of the elastomer adhesives

By virtue of the short relaxation time, the elastomer adhesives show an excellent self-recovery ability. As displayed in Figure S3a, the successive cyclic load-unloading stress-strain curves exhibit that the *f* = 0.4 elastomer does not show a typical Mullins effect and can instantly recover the hysteresis upon the next loading-unloading cycle.

We also evaluate the self-recovery ability of the elastomer at a fixed strain of 400%. The results show that the elastomer can rapidly recover most of the hysteresis in a short time (Figure S3b). The recovery ratio approaches 100% (Figure S3c).

The high mobility also allows polymer chains to diffuse into each other across the fracture area when the elastomer is cut, giving rise to a self-healing ability.[1, 2] As shown in Figure S4a, the mechanical properties of the cut sample gradually recover as the healing time increases. By comparing the work of extension at fracture (W_{extf}) of the original and healing samples, we can define the healing efficiency. As shown in Figure S4b, the healing efficiency increases with the healing time, due to the increase of diffusion time. Meanwhile, the healing process can be accelerated by increasing the temperature, which results in an augment in the mobility of polymer chains. As exhibited in Figure S4c, after healing at 80 °C for 0.5 h, the healing efficiency of the f = 0.4 elastomer can approach 60%. As a demonstration, we show a typical self-healing process of the f = 0.4 elastomer in Figure S4e. The cut gradually disappears after the sample is healed for 24 h at room temperature. The healed sample can still be highly deformed by elongation.

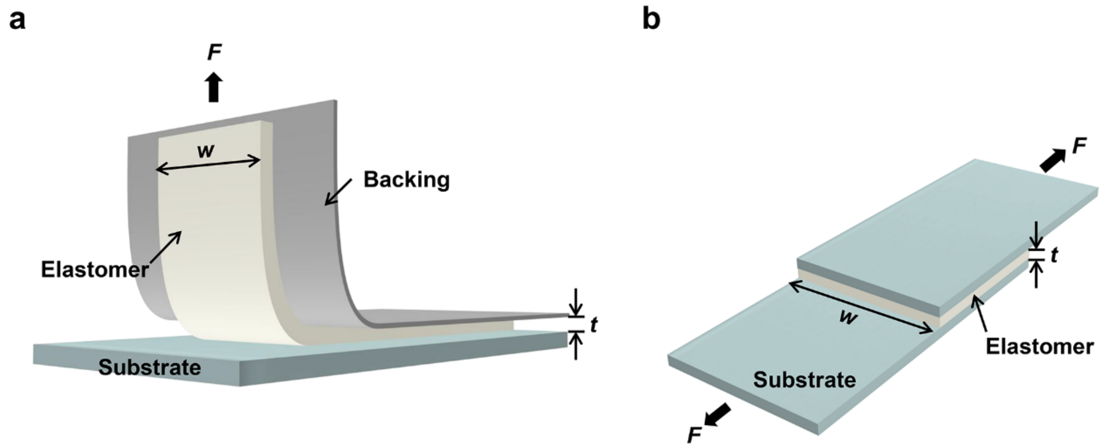

Figure S5. a) Experimental setup of the 90-degree peeling test. A stiff backing is attached to the top side of the elastomer to prevent the elongation of the elastomer during interfacial separation. b) Experimental setup of the lap shear test.

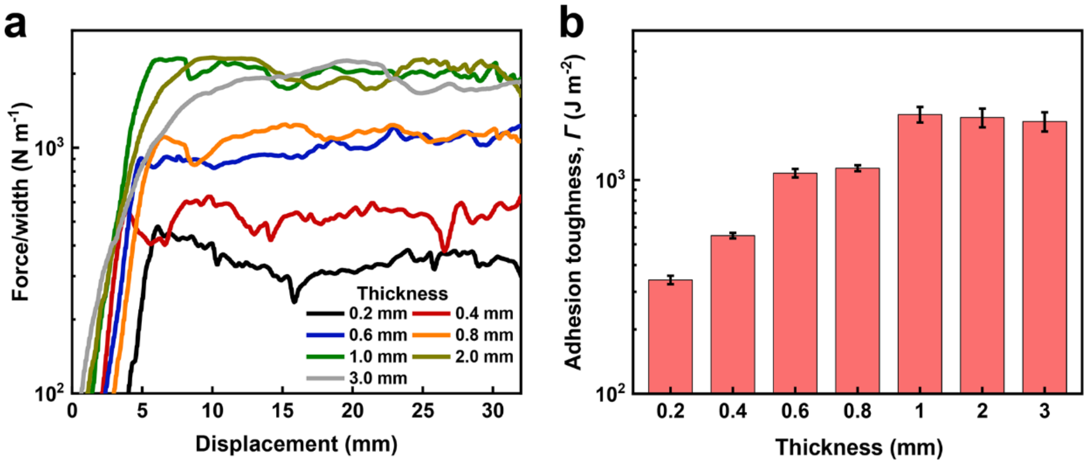

Figure S6. a) Influence of sample thickness on the force/width-displacement curves of the f = 0.4 elastomer during 90-degree peeling tests. The testing velocity is fixed as 50 mm min^{-1}. b) Calculated adhesion toughness of the f = 0.4 elastomer with different thicknesses.

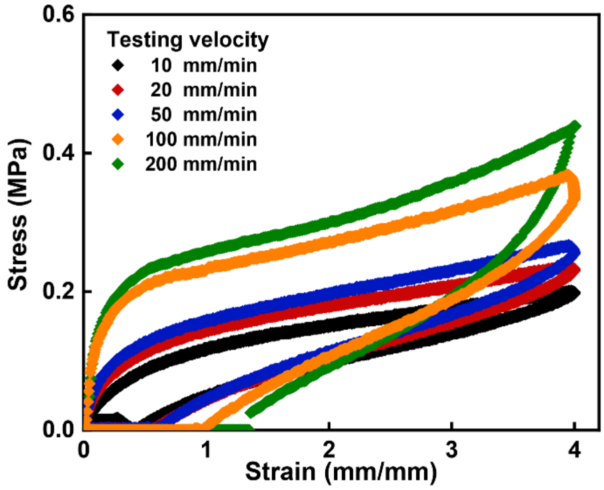

Figure S7. Rate-dependent mechanical performance of the f = 0.4 elastomer reflected by the cyclic loading-unloading test at a fixed strain of 400%. The elastomer shows increased stress, Young's modulus, and hysteresis with the augment of testing velocity.

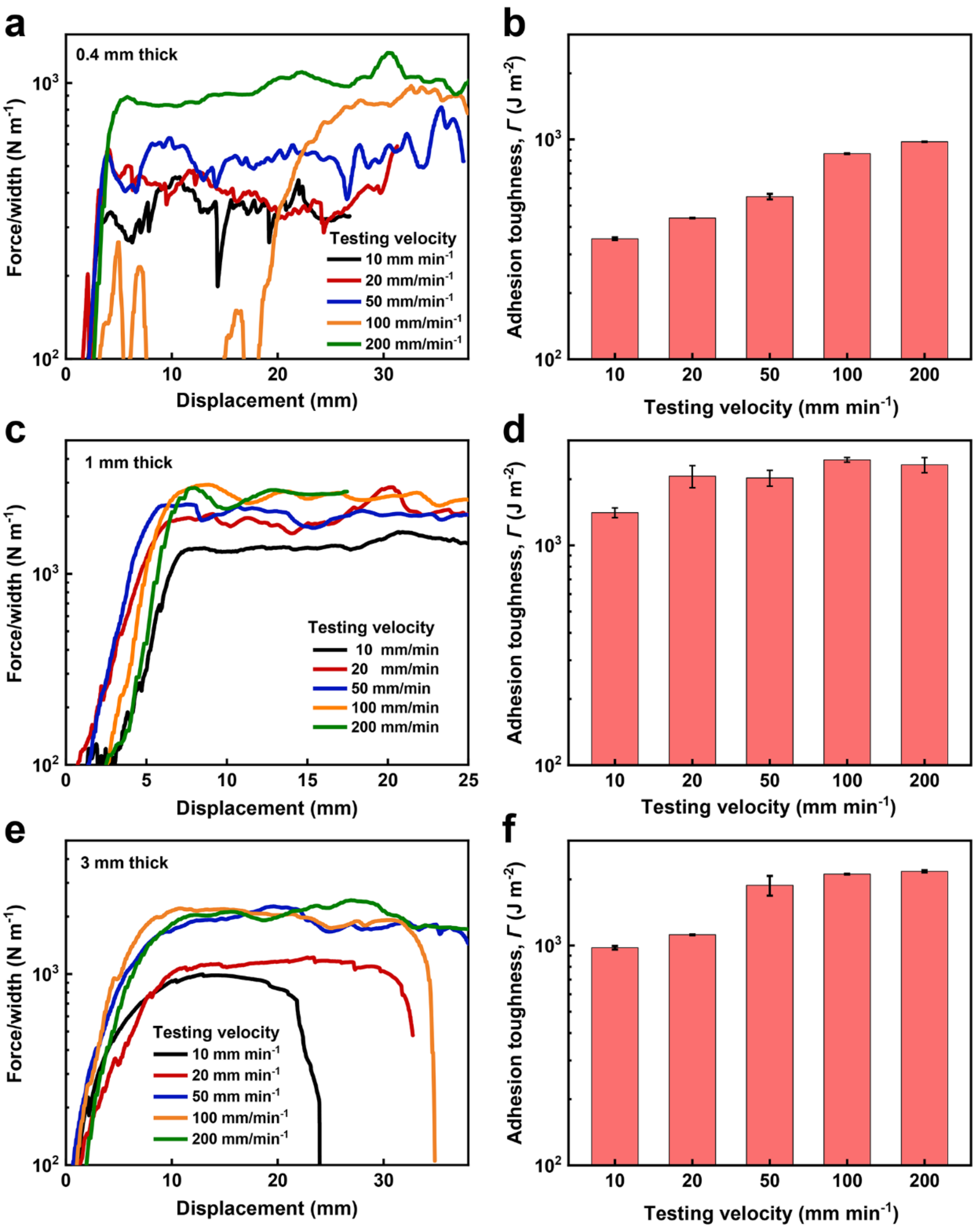

Figure S8. a) Influence of testing velocity on the force/width-displacement curves of the f = 0.4 elastomer with a thickness of 0.4 mm during 90-degree peeling tests. b) Calculated adhesion toughness of the 0.4 mm thick f = 0.4 elastomer under different testing velocities. c) Influence of testing velocity on the force/width-displacement curves of the f = 0.4 elastomer with a thickness of 1 mm during 90-degree peeling tests. d) Calculated adhesion toughness of the 0.4 mm thick f = 1 elastomer under different testing velocities. e) Influence of testing velocity on the force/width-displacement curves of the f = 0.4 elastomer with a thickness of 3 mm during 90-degree peeling tests. b) Calculated adhesion toughness of the 3 mm thick f = 0.4 elastomer under different testing velocities.

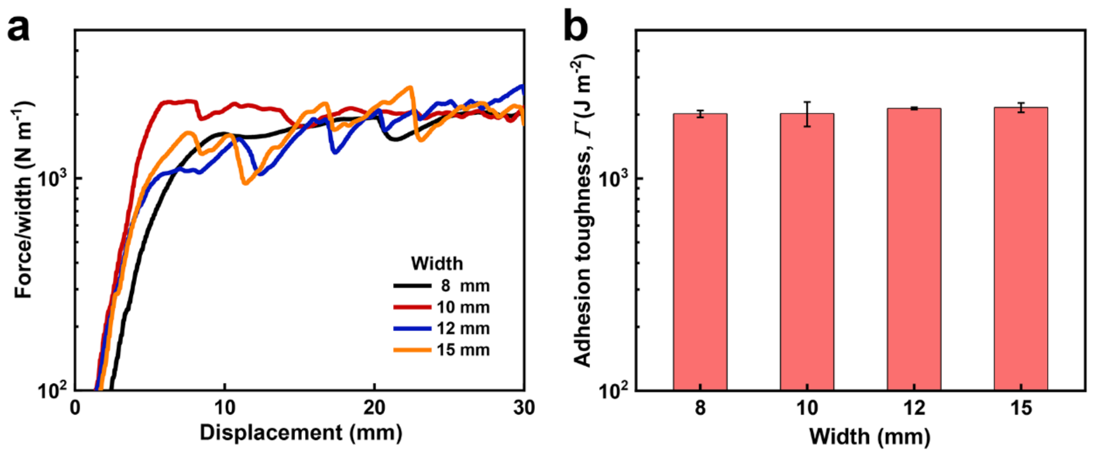

Figure S9. a) Influence of sample width on the force/width-displacement curves of the *f* = 0.4 elastomer during 90-degree peeling tests. The testing velocity is fixed as 50 mm min^{-1}. b) Calculated adhesion toughness of the *f* = 0.4 elastomer with different widths.

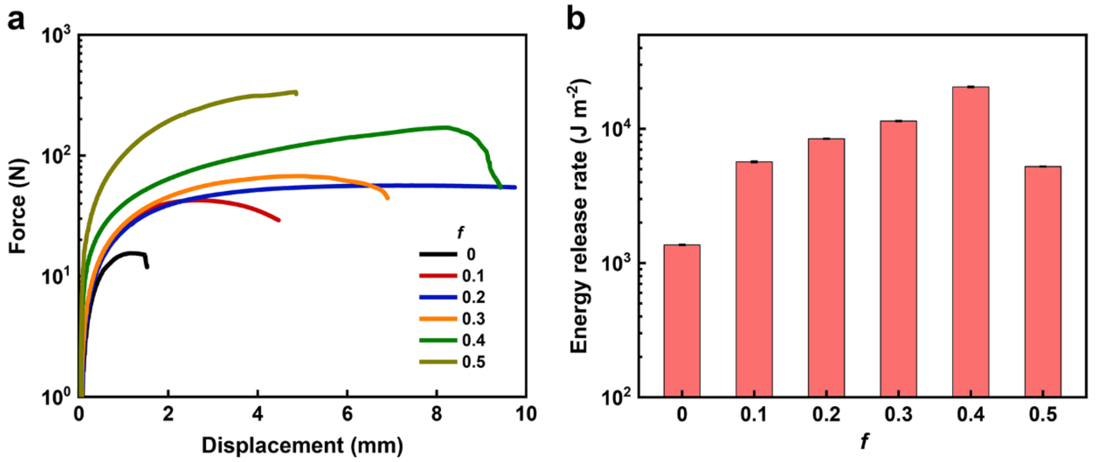

Figure S10. a) Force-displacement curves of the P(BA-co-IBA) elastomers with varied *f* during the lap shear test. b) Calculated energy release rate of the elastomers with varied *f*.

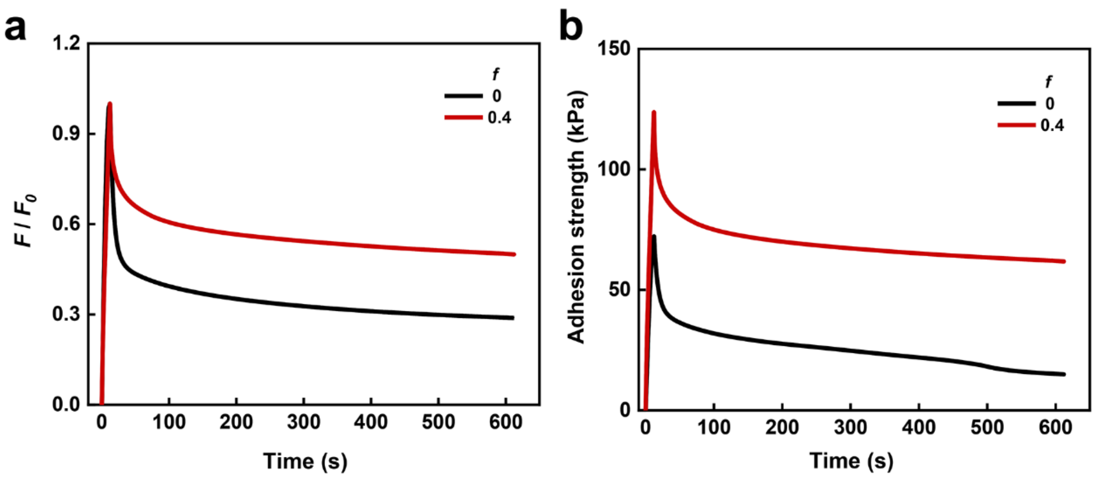

Figure S11. Relaxation test on the $f = 0$ and $f = 0.4$ elastomers using the experimental setup of the lap shear test. a) The evolution of adhesion force for the two elastomers. b) The evolution of adhesion strength for the two elastomers.

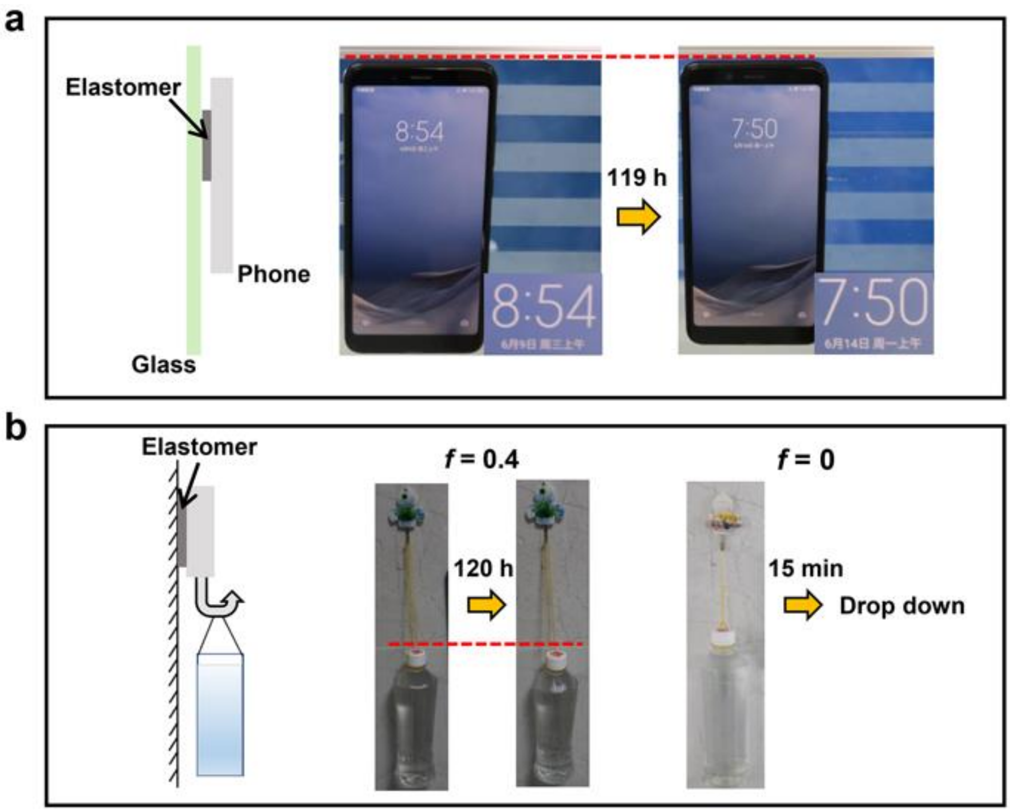

Figure S12. Utilization of the $f = 0.4$ elastomer as a strong and long-lasting adhesive in daily life. a) Using the elastomer to adhere a phone to glass using the $f = 0.4$ elastomer. The position of the phone does not change for 119 h. b) Using the elastomer to glue a hook that sustains a container with 500 mL of water. The hook that uses the $f = 0.4$ elastomer as the adhesive hardly slides down for 120 h, while the hold using $f = 0$ elastomer as the adhesive drops down after 15 min.

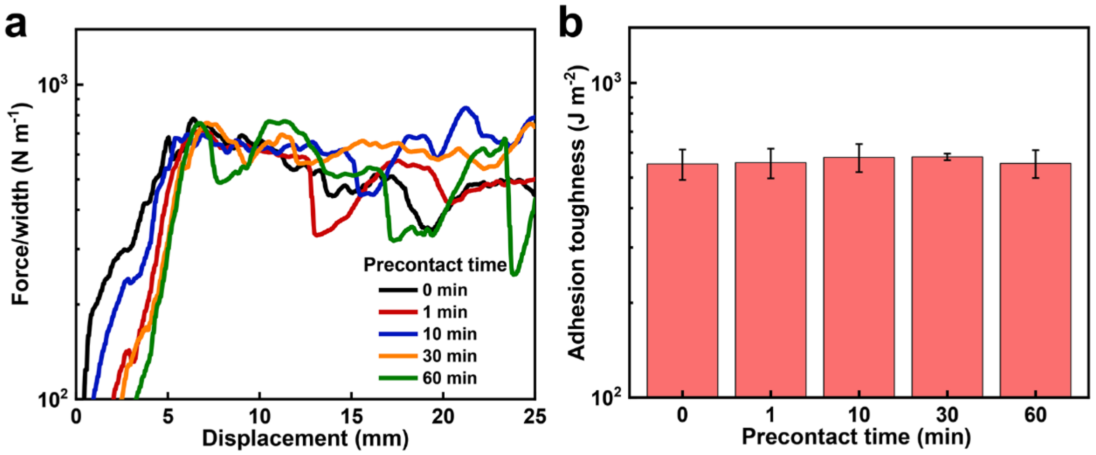

Figure S13. a) Force/width versus displacement curves of the f = 0.5 elastomer that adheres to glass under different precontact times during the 90-degree peeling test. b) Adhesion toughness of the f = 0.5 elastomer under different precontact times.

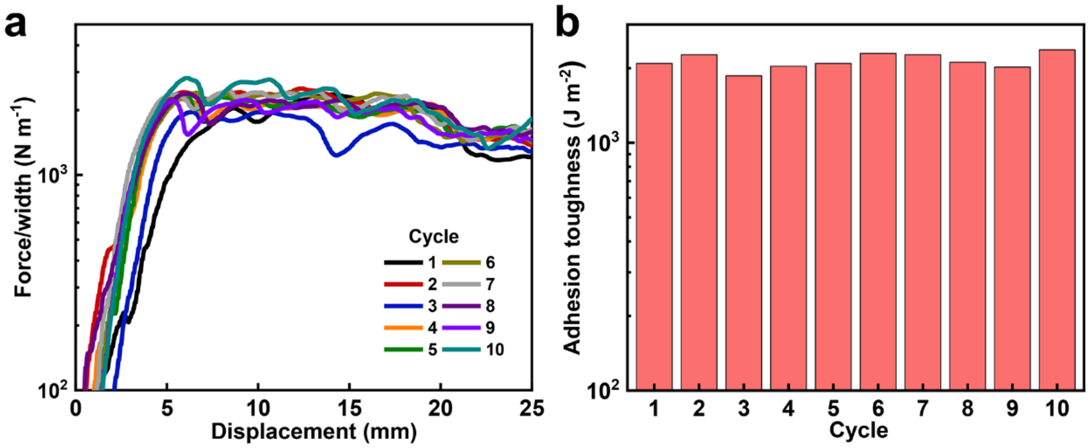

Figure S14. a) Force/width versus displacement curves of the f = 0.4 elastomer at different adhesion cycles (1 to 10). b) Adhesion toughness of the f = 0.4 elastomer at different adhesion cycles.

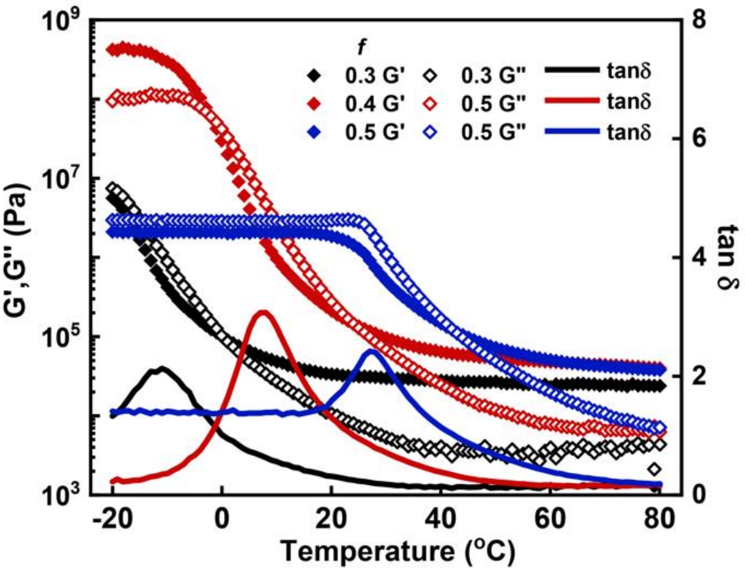

Figure S15. Temperature scanning of P(BA-co-IBA) elastomers at varied *f* (*f* = 0.3, 0.4, 0.5) in rheological tests.

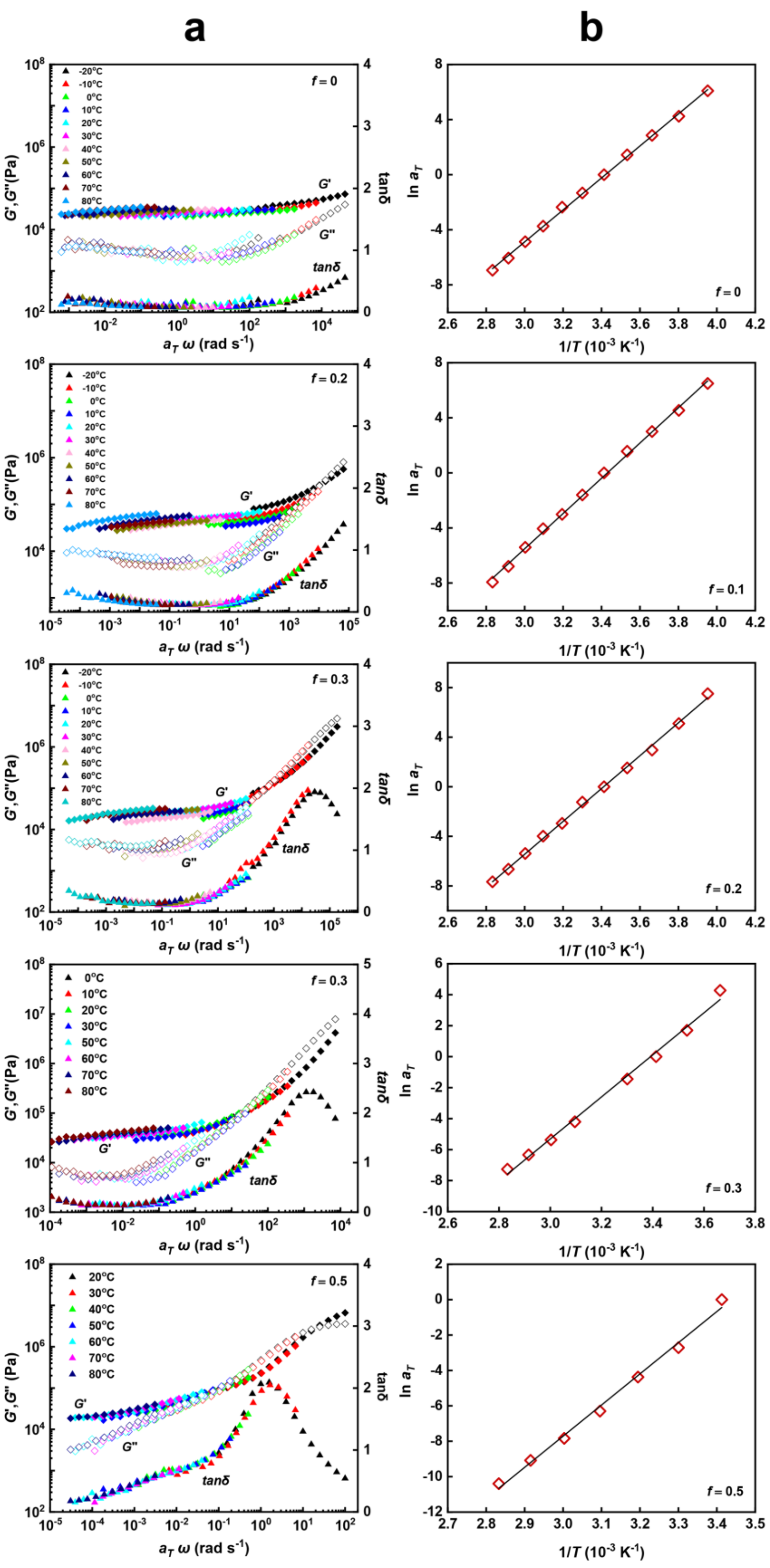

Figure S16. a) Master curves of G', G'', and $tan\delta$ of the P(BA-co-IBA) elastomers with varied f (0, 0.1, 0.2, 0.3, 0.5) with 20 °C as the reference temperature. b) Arrhenius plot for the temperature-dependent shift factors (a_T) for the P(BA-co-IBA) elastomers with varied f (f = 0, 0.1, 0.2, 0.3, 0.5). The apparent activation energy (E_a) is calculated from the slope of the curve.

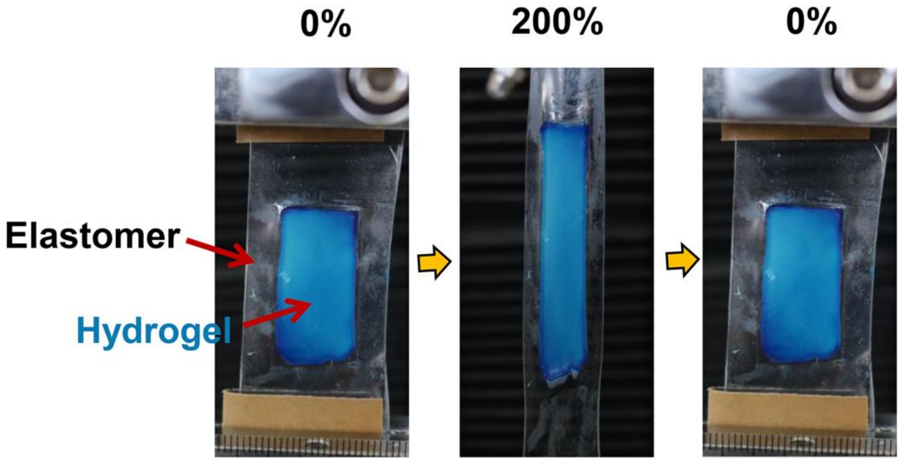

Figure S17. Demonstration of the tough adhesion between the gel layer and the elastomer layer in the multilayered sensor. The hydrogel and the elastomer deform simultaneously when the sensor is significantly stretched. After releasing the sensor, no delamination is observed between the gel and the elastomer.

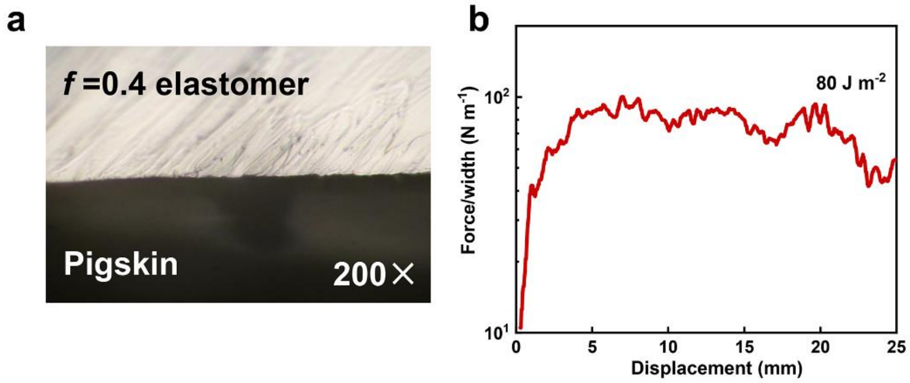

Figure S18. a) Optical image of the interface between the f = 0.4 elastomer and a piece of pigskin. b) Force/width-displacement curve of the f = 0.4 elastomer that is adhered to the pigskin during the 90-degree peeling test.

References

[1] L. Chen, T. L. Sun, K. Cui, D. R. King, T. Kurokawa, Y. Saruwatari, J. P. Gong, *J. Mater. Chem. A* **2019**, *7*, 17334.

[2] W. Urban Marek, D. Davydovich, Y. Yang, T. Demir, Y. Zhang, L. Casabianca, *Science* **2018**, *362*, 220.
